# Stem cell origin differently affects bone tissue engineering strategies

**DOI:** 10.3389/fphys.2015.00266

**Published:** 2015-09-24

**Authors:** Monica Mattioli-Belmonte, Gabriella Teti, Viviana Salvatore, Stefano Focaroli, Monia Orciani, Manuela Dicarlo, Milena Fini, Giovanna Orsini, Roberto Di Primio, Mirella Falconi

**Affiliations:** ^1^Department of Clinical and Molecular Sciences, Università Politecnica delle MarcheAncona, Italy; ^2^Department of Biomedical and Neuromotor Sciences, University of BolognaBologna, Italy; ^3^Laboratory of Preclinical and Surgical Studies, Rizzoli Orthopaedic InstituteBologna, Italy; ^4^Department of Clinical Sciences and Stomatology, Università Politecnica delle MarcheAncona, Italy

**Keywords:** PDPCs, ASCs, PDL-SCs, tissue engineering, q-RT-PCR, SEM, TEM

## Abstract

Bone tissue engineering approaches are encouraging for the improvement of conventional bone grafting technique drawbacks. Thanks to their self-renewal and multi-lineage differentiation ability, stem cells are one of the major actors in tissue engineering approaches, and among these adult mesenchymal stem cells (MSCs) hold a great promise for regenerative medicine strategies. Bone marrow MSCs (BM-MSCs) are the first- identified and well-recognized stem cell population used in bone tissue engineering. Nevertheless, several factors hamper BM-MSC clinical application and subsequently, new stem cell sources have been investigated for these purposes. The fruitful selection and combination of tissue engineered scaffold, progenitor cells, and physiologic signaling molecules allowed the surgeon to reconstruct the missing natural tissue. On the basis of these considerations, we analyzed the capability of two different scaffolds, planned for osteochondral tissue regeneration, to modulate differentiation of adult stem cells of dissimilar local sources (i.e., periodontal ligament, maxillary periosteum) as well as adipose-derived stem cells (ASCs), in view of possible craniofacial tissue engineering strategies. We demonstrated that cells are differently committed toward the osteoblastic phenotype and therefore, taking into account their specific features, they could be intriguing cell sources in different stem cell-based bone/periodontal tissue regeneration approaches.

## Introduction

Reconstruction of large bone and/or complex craniofacial defects is a clinical challenge in situations of injury, congenital defects or disease. The use of cell-based therapies represents one of the most advanced methods to enhance the regenerative response for bone wound healing. As suggested by Giannoudis et al. (2007), in addition to 3D dimensional structures, mechanical, and/or physical signals, cell type as well as environmental bioactive factors are critical to direct tissue repair and regeneration. Both somatic and stem cells have been adopted in the treatment of complex osseous defects and, among these, mesenchymal stem cells (MSCs) held the greatest promise for regenerative therapies in the skeletal system. MSCs are multipotent and self-renewing cells owning endogenous functions for tissue renewal and repair within their individual local tissues (van der Kooy and Weiss, 2000). The term MSCs was originally generated considering a theoretical common progenitor of a wide range of “mesenchymal” tissues (Caplan, 1991) and these cells are commonly identified because of their surface phenotype and *in vitro* ability to differentiate into specific lineages (Dominici et al., 2006). Even though it has been widely accepted that MSCs reside ubiquitously throughout a variety of post-natal tissues and organs (Crisan et al., 2008), this concept undergoes to strong criticism for the missing of an essential *in vivo* experimental support (Bianco et al., 2013). Moreover, recent literature suggests that not all MSCs are certainly created equal in their differential and proliferative capacities, or in their capability to respond to external influences (i.e., microenvironment). Cells harvested from different sources may in fact show phenotypic heterogeneity and dissimilar *in vivo* results after transplantation (Rebelatto et al., 2008). For researchers investigating stem cell-based tissue engineering, it is essential to select the most appropriate type of MSCs source naturally suited to obtain a more efficient treatment for the regeneration of injured skeletal tissues of different anatomical districts.

To this aim, we tested the cross-talk between 3D porous scaffolds and MSCs derived from different adult human tissues. The choice of the different MSCs population harvesting sites (i.e., periosteum, periodontal ligament and adipose tissue) was based on their possible regenerative medicine applications in complex craniofacial lesions.

Periosteum derived precursor cells (PDPCs) reside in the inner “cambium layer” of periosteum which is a connective structure comprised of 2 layers that covers the external surface of bone (Roberts et al., 2015). Specifically, besides osteoblasts and bone lining cells, the inner layer has adult mesenchymal skeletal progenitor cells, smaller and more isodiametric fibroblasts, and sympathetic nerves that make periosteum a structure with regenerative capacity (Ferretti and Mattioli-Belmonte, 2014a; Lin et al., 2014). Periodontal ligament stem cells (PDL-SCs) that reside at the perivascular regions possess characteristics of MSCs and are a promising tool for periodontal regeneration (Zhu and Liang, 2015). At last, Adipose-derived Stem Cells (ASCs) were found to be more suitable in clinical application in comparison with those derived by bone marrow for higher stem cells harvest from lipoaspirates, faster cell proliferation and less discomfort and morbidities during collecting procedure. However, conflicting results on their osteogenic capacity is now still debated (Liao and Chien, 2014).

In this study, PDPCs, PDL-SCs, and ASCs were seeded on two kinds of gelatin/genipin scaffolds for 14 and 21 days and cultured in appropriate differentiating media in order to mimic a chondrogenic or osteogenic microenvironment. Cell proliferation assay, light microscopy, transmission (TEM) and scanning (SEM) electron microscopies and qRT-PCR were carried out to evaluate cell viability, morphological and functional changes induced by cell/scaffold interaction.

## Materials and methods

### Cell culture

#### Periosteal derived progenitor cells (PDPCs)

PDPCs were obtained from maxillary periosteal tissue of four subjects undergoing routine oral surgery (mean age 34 years), after the obtainment of their informed consent. As previously described (Ferretti et al., 2012, 2014b), tissue was washed in Dulbecco's Phosphate-Buffered Saline (D-PBS) lacking in Ca^2+^ and Mg^2+^, minced into small pieces (4–9 mm^2^) and then placed in a 100 mm Petri dish in Dulbecco's Modified Eagle Medium: Nutrient Mixture F-12 (DMEM/F-12, Sigma-Aldrich, Milan, Italy) supplemented with 10% Fetal Bovine Serum (FBS) and 1% penicillin-streptomycin (100 U/ml), all from GIBCO® (Life Technologies Corporation, USA). DMEM-F12 provides optimal and appropriate culture conditions for MSCs isolation and *ex vivo* expansion, preserving the correct morphology, population doubling time and immunophenotype (Pal et al., 2009). Periosteum explants were positioned with their cambium side placed against the dishes to allow cell adhesion. Petri dishes were incubated at 37°C in a humidified, CO_2_-controlled (5%) incubator. Medium was changed twice a week. As soon as cells migrating from the explants reached 50% of confluence, they were collected by treatment with 0.25% trypsin/1 mM EDTA (Sigma-Aldrich, Milan, Italy) and subcultured at 1:3 dilutions under the same culture condition. Cells were used at the 3rd passage to assess their MSC phenotype and their ability to differentiate into mesenchymal lineages.

#### Periodontal ligament stem cells (PDL-SCs)

Periodontal ligaments tissues were obtained from extracted human molars, of 10 healthy volunteers aged 16–30 years. Written informed consent was obtained from all donors. As previously described (Orciani et al., 2012), periodontal ligaments tissues were cut in small pieces and cultured in Petri dishes in Dulbecco's Modified Eagle Medium: Nutrient Mixture F-12 (DMEM/F-12) (Gibco, Life Technologies, Milan, Italy) containing 10% FBS, penicillin (100 U/mL) and streptomycin (100 μg/mL) (Gibco, Life Technologies, Milan, Italy). Cells were grown until 50% confluence was reached, then they were harvested by treatment with 0.25% trypsin/1 mM EDTA (Gibco, Life Technologies, Milan, Italy) and re-plated at 1:2 dilutions under the same culture condition. Cells were used at the 3rd passage for phenotypic characterization and differentiation.

#### Adipose derived stem cells (ASCs)

StemPro® Human ASCs were purchased from Life Technologies Corporation (Monza, Italy). Cells were grown in MesenPRO RS™ Basal Medium (Life Technologies, Monza, Italy) supplemented with MesenPRO RS™ Growth Supplement (Life Technologies, Milan, Italy) according to the manufacture's suggestions. ASCs were expanded to 4–5 passages before they lost the capacity to grow or differentiate into all potential phenotypes.

### Cell characterization

According to The International Society for Cellular Therapy for identification of human MSCs (Dominici et al., 2006), PDPCs, PDL-SCs, and ASCs were analyzed by flow cytometry and subjected to differentiation into mesenchymal lineages.

For immunophenotyping, 2.5 × 10^5^ cells were washed with D-PBS and then stained for 45 min with the following antibodies: fluorescein isothiocyanate-(FITC)-labeled mouse anti-human CD90 (Stem Cell Technologies—Milan, Italy), CD105, CD14, CD19 (Diaclone, France), and R-phycoerythrin-(PE)-labeled mouse anti-human CD34, CD45 (Diaclone, France), CD73 (Becton Dickinson) and anti HLA-DR purchased from Diaclone. Control for FITC or PE coupled antibodies was an isotypic mouse IgG1.

Flow cytometric analysis was performed on a FACSCalibur system (Becton Dickinson, CA, USA) using CellQuest software (Becton Dickinson). To evaluate fibroblastic contamination, we tested FITC-labeled mouse anti-human CD9 monoclonal antibody (ImmunoTools GmbH, Germany) (Halfon et al., 2011).

Cell differentiation into osteoblasts, adipocytes and chondrocytes was evaluated using STEMPRO® Osteogenesis, Adipogenesis and Chondrogenesis Kits (Life Technologies Corporation, USA) respectively. Cells cultured in DMEM/F-12 with 10%FBS were used as negative controls.

For osteoblastic differentiation, cells were plated at a density of 4.5 × 10^4^ cells with appropriate medium for 10 days, refreshing the medium every 2 days. In order to assess the osteoblastic differentiation von Kossa and Alkaline phosphatise (ALP) stainings were performed. For von Kossa stain, cells were fixed with 4% Paraformaldehyde (PFA) for 15 min at room temperature (RT) and incubated with 1% silver nitrate solution under UV light for 20 min at RT. Unreacted silver was removed with 5% sodium thiosulfate for 5 min. For ALP staining, cells were fixed with 4% PFA for 15 min RT and washed in 100 mM Tris-HCl pH 9.5, 100 mM NaCl and 10 mM MgCl2 buffer for 10 min RT. Cells were then stained with fast 5-bromo-4-chloro-3-indolyl phosphate and nitroblue tetrazolium alkaline phosphate substrate (Sigma-Aldrich, Milan, Italy) for 10 min and rinsed in dH_2_O. Reaction was observed with a light microscope (Nikon Eclipse 600, Nikon, Milan, Italy).

For adipogenic differentiation 9 × 10^4^ cells were seeded and treated with the appropriate medium for 15 days, changing the media twice a week. Differentiation was assessed by Oil Red staining and CD36 immunoreaction. Briefly, cells fixed in 4% PFA were exposed to Oil Red O solution (0.5% in 100% isopropyl alcohol) for 20 min RT, cleared with isopropanol 60% and finally washed in dH_2_O. For the detection of CD36 positivity, PDPCs were incubated with monoclonal anti-CD36 (ImmunoTools GmbH, Friesoythe, Germany) diluted 1:100. The reaction was visualized using the streptavidin-biotin-peroxidase technique (DAKO LSAB+/HRP peroxidase kit; Dako SpA, Milano, Italy). Cells were incubated with 3,3-diaminobenzidine-DAB (10 mg diaminobenzidine in 15 ml 0.05 M Tris buffer, pH 7.6 and 12 μl hydrogen peroxide 30%) and counterstained with Mayer's haematoxylin (Bio-Optica SpA, Milan, Italy). Reaction was examined with a light microscope (Nikon Eclipse 600).

For chondrogenic differentiation, cells were cultured in pellet culture system. For the preparation of each pellet, aliquots of 1 × 10^6^ cells in 1 ml of appropriate medium were spun down at 1200 rpm for 5 min. Pellets were cultured for 20 days changing the medium twice a week. Pellets were then fixed in 4% PFA, paraffin embedded and sectioned. Sections were exposed to a solutions of Alcian Blue pH 1 (Bio-Optica) for 20 min RT or Safranin-O (0.1 g in EtOH 100%, working dilution 1: 2 dH_2_O) for 5 min RT and observed with a light microscope (Nikon Eclipse 600).

### Scaffold preparation

3D Gelatin (G) scaffolds (GEL) were obtained using type A gelatin from pig skin cross-linked with genipin as previously described (Panzavolta et al., 2013). Briefly, following the addition of genipin, the foam assumed a blue color due to the binding with primary amino-groups of the gelatin. For the preparation of scaffolds containing hydroxyapatite (HA), 10 wt% powders was added to 140 ml of a gelatin solution in order to obtain a suspension (G/HA). The suspension was maintained under constant mechanical stirring and subsequently cross-linked with genipin. The samples were then allowed to jellify in an oven, washed in a glycine aqueous and finally frozen in liquid N_2_ and freeze-dried (GEL/HA scaffold).

Before seeding the scaffolds were sterilized in 70% ethyl alcohol solution (ETOH; Sigma-Aldrich, Milan, Italy) for 2 h, washed two times in PBS (GIBCO) for 30 min and placed under UV 15 min for each side. In order to improve cell adhesion, scaffolds were then conditioned overnight in suitable media at 5% CO2, 37°C. The media were then discarded and scaffolds considered ready for seeding. Cells were detached using 0.25% trypsin in 1 mM ethylene-diamine- tetracetic -acid (EDTA, Sigma-Aldrich, Milan; Italy) and seeded at a density of 1 × 10^4^ cell/cm^3^ by applying 50 μL of cell suspension on the samples placed at 37°C for 30 min in a humified chamber, in order to avoid the slip down of cells. After 1.5 ml of STEMPRO® Osteogenesis or STEMPRO® Chondrogenesis medium was added to cover G/HA or G scaffolds placed in Corning® ultra-low attachment multiwell plates, respectively. Cells were cultured for 7, 14, and 21 days.

### MTT (3-dimethylthiazol-2,5-diphenyltetrazolium bromide) viability assay

**Figure 1:**
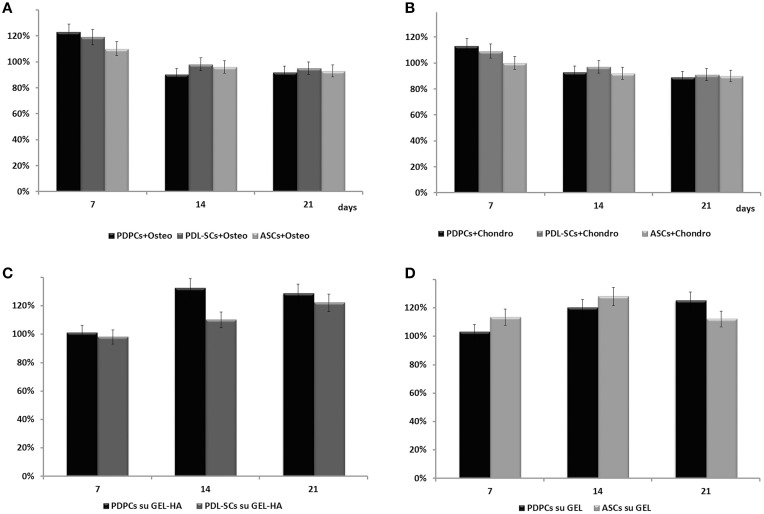
**Histograms depict MTT viability test performed in PDPCs, PDL-SCs, and ASCs cultured in tissue culture plates for 7, 14, and 21 days in osteogenic (A) or chondrogenic (B) medium**. Data are expressed respectively as percentage over PDPCs, PDL-SCs, and ASCs cultured for 7, 14, and 21 days in undifferentiating medium; **(C)** PDPCs and PDL-SCs cultured on GEL/HA scaffolds for 7, 14, and 21 days in osteogenic medium. Data are expressed respectively as percentage over PDPCs and PDL-SCs cultured in osteogenic media in tissue culture plates in osteogenic media. **(D)** PDPCs and ASCs cultured on GEL scaffolds for 7, 14, and 21 days in chondrogenic medium. Mean values ± SD are reported.

Cell viability was assessed in PDPCs, ASCs, and PDL-SCs after 7, 14, and 21 days of culture in basal and differentiating osteogenic or chondrogenic media. Cell viability was also evaluated in cells cultured on GEL and GEL/HA scaffolds. Briefly, after removing the culture media, 200 μL of MTT (3-dimethylthiazol-2,5-diiphenyltetrazolium bromide, 135038 Sigma-Aldrich) solution (5 mg/mL in phenol red-free DMEM) and 1.8 mL DMEM were added to the multi-well plates and incubated at 37°C for 3 h. After discarding the supernatants, 2 mL of solvent (4%HCl 1N in isopropanol absolute) were added to dissolve the dark blue formazan crystals and quantify them spectrophotometrically measuring the absorbance at 570 nm (Secoman, Anthelie light, version 3.8, Contardi, Italy). Data were expressed as percentage over the respective control culture (see Figure 1 legend).

### Scanning electron microscopy (SEM)

Samples from cell culture tests were fixed in 2% glutaraldehyde (Sigma-Aldrich) in 0.1 M cacodylate buffer (pH 7.4, Sigma-Aldrich), post-fixed in 1% osmium tetroxide (Sigma, Milan, Italy), dehydrated in increasing ethanol (Sigma-Aldrich) concentrations (25, 50, 70, 80, and 100%), CPD-dried, mounted on aluminum stubs, gold-sputtered by the Edwards Sputter Coater B150S equipment and observed with a Philips XL 20 SEM (FEI Italia SRL, Milan, Italy) microscope.

### Transmission electron microscopy (TEM)

Cells cultured on scaffolds were fixed in 2.5% glutaraldehyde in 0.1 M cacodylate buffer for 2 h at 4°C and post-fixed in 1% Osmium tetroxide in 0.1 M cacodylate buffer for 30 min at room temperature. Samples were dehydrated in graded ethanol and finally infiltrated and embedded in RL London White (Fluka, Sigma Aldrich, St. Louis, Missouri, USA). 100 nm ultra-thin sections were cut using a Diatome (Diatome, Hatfield, PA, USA) diamond knife on a NOVA LKB Ultratome. Sections were picked up on nickel grids and stained with alcoholic uranyl acetate and Reynold's lead citrate. Ultrastructural examination was performed using the Philips CM10 Transmission Microscope (FEI Company, Eindhoven, The Netherlands). Images were recorded by Megaview III digital camera (FEI Company, Eindhoven, The Netherlands).

### Quantitative real-time polymerase chain reaction (qRT-PCR)

#### RNA extraction, quantitation and reverse transcription

Total RNA was extracted from cells with TRIzol® Reagent (Life Technologies, Milan, Italy) according to the manufacturer's protocol. RNA samples were quantified by measuring their absorbance at 260 nm (bioPhotometer plus, Eppendorf GmbH, Germany). The Go Script™ RT System (Promega Corporation—Italy) was used to reverse transcribe 1 μg of total RNA in a 20-μL reaction volume. cDNA neo-synthesized was stored at −20°C.

#### qRT- PCR

Real-time PCR was carried out in white plastic-ware with a Mastercycler Realplex2 thermocycler (Eppendorf GmbH, Germany) using the SsoFast™ EvaGreen® Supermix 1X. All PCR assays contained 1 μL of cDNA (corresponding to 50 ng of total RNA template) in a 10-μL reaction volume.

The following program was used for amplification: enzyme activation for 30 s at 95°C, followed by 40 cycles of denaturation for 5 s at 95°C, annealing and extension at 60°C for 20 s. Each primer was used at a 200 nM final concentration. Primer sequences were designed by Primer 3 (v. 0.4.0) software and their specificity was tested by BLAST Assembled RefSeq Genomes in order to avoid any appreciable homology to pseudo-genes or other unexpected targets (Table 1). In each assay, the mRNA of both reference genes and each gene of interest were measured simultaneously under equal conditions. Primers showed the same amplification efficiency. Melting curve analysis furthermore confirmed the specificity of qRT-PCR reactions.

**Table 1:** **Analysed genes description**.

**Gene**	**Detected transcript**	**Primer forward (5′->3′)**	**Primer reverse (5′->3′)**	**Annealing T (°C)**
RUNX2	NM_004348.3	CTCGTCCGCACCGACAGCC	TACCTCTCCGAGGGCTACCACC	60
BMP2	NM_001200.2	CCAGCCGAGCCAACACTGTGC	TCTCCGGGTTGTTTTCCCACTCG	60
SPARC	NM_003118.3	CCTGAGGCTGTAACTGAGAGAAAG	GTGGGAGGGGAAACAAGAAGATAA	65
BGLAP	NM_199173	GACTGTGACGAGTTGGCTGA	GCCCACAGATTCCTCTTCTG	64
SOX9	NM_000346	GAGGAAGTCGGTGAAGAACG	ATCGAAGGTCTCGATGTTGG	65
Type II Collagen	NM_001248899	GGCAATAGCAGGTTCACGTACA	CGATAACAGTCTTGCCCCACTT	60
GAPDH ^*^	NM_002046.3	AGCCACATCGCTCAGACAC	GCCCAATACGACCAAATCC	60
GUSB^*^	NM_000181.2	AAACGATTGCAGGGTTTCAC	TCTCGTCGGTGACTGTTCA	81

Runx2, runt-related transcription factor 2; Bmp2, bone morphogenetic protein 2; Sparc, Osteonectin; Bglap, osteocalcin; Sox9, SRY (sex determining region Y)-box 9; Gusb, beta glucuronidase; Gapdh, glyceraldehyde-3-phosphate dehydrogenase.

* Reference genes.

#### Quantification of mRNA expression

Real-time PCR reactions were performed in triplicate and Ct values of reference genes were used to normalize cellular mRNA data. In this instance, normalization involved the ratio of mRNA concentrations of specific genes of interest (as mentioned above) to that corresponding to Ct medium values for glyceraldehyde-3-phosphate dehydrogenase (GAPDH) and beta glucuronidase (GUSB) (Ragni et al., 2013). Data were expressed as gene relative expression (2^−ΔCt^). Furthermore, in order to highlight the effect of mechanical stimuli on cells, the ΔΔCt method for the evaluation of Fold-Change was employed and cells seeded on plastic were used as an internal control. The relative amount of each mRNA was calculated using the comparative threshold (Ct) method with ΔCt = Ct(mRNA) − Ct(GAPDH) and relative quantification of mRNA expression was calculated with the 2^−ΔΔCt^ method (Livak and Schmittgen, 2001). The qPCR efficiency in all our experiments was more than 90%, as the difference between the actual and theoretical (100%) efficiencies would result in an underestimation of the mRNA concentration of all analyzed samples.

Data in histograms were expressed as fold-regulation that represents fold-change results in a biologically meaningful way. In particular, the fold-regulation is equal to the fold-change (2^−ΔΔCt^) for fold-change values greater than one, which indicate an up-regulation. Fold-change values less than one indicate a down-regulation: in this case the fold-regulation is the negative inverse of the fold-change (−1/2^−ΔΔCt^).

### Statistical analysis

Mean and standard deviation of three different experiments are reported. Data were analyzed by One-Way ANOVA, Student-Newman-Keuls's and Student's T tests. Statistical significance was tested at *p* < 0.05

## Results

### Cell characterization

PDPCs, PDL-SCs, and ASCs were all plastic-adherent under standard culture conditions with a fibroblastic, spindle-shape appearance. All cell populations expressed stromal surface markers CD73, CD90, and CD105 and were negative for hematopoietic lineage markers CD45, HLA-DR, CD14, CD19, and CD34 in agreement with the criteria of the International Society for Cell Therapy (Dominici et al., 2006). Moreover they were able to differentiate into all the mesenchymal lineages (data not shown).

### MTT viability test

A significant decrease in cell viability was observed in all tested cells after 14 days of culture in osteogenic or chondrogenic medium. No changes were detected between 14 and 21 days of culture (Figures 1A,B). These results well-matched with cell differentiation.

PDPCs cultured on GEL/HA scaffolds showed a significant increase of cell viability up to 21 days. A similar behavior was detected also for PDL-SCs even though at a lower extent (Figure 1C).

As far as cell seeded on GEL scaffold, PDPCs showed a trend similar to that observed on GEL/HA, whilst ASCs exhibited an increase in cell viability after 14 days of culture and then a decrease at 21 days (Figure 1D).

### SEM

**Figure 2:**
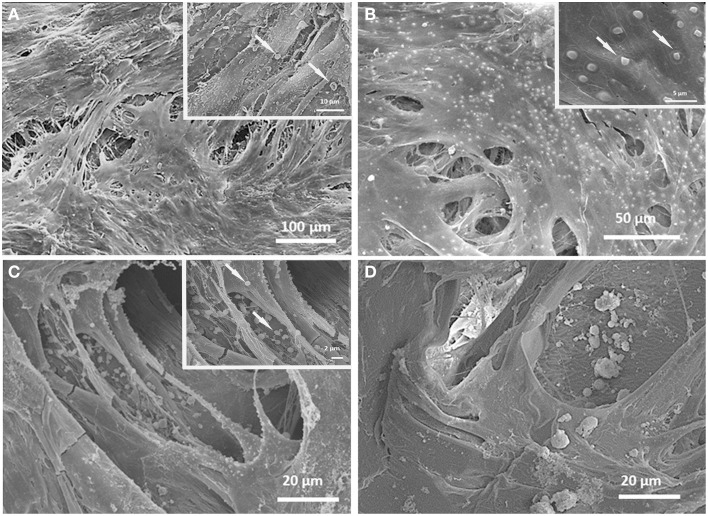
**SEM micrographs of cells cultured on GEL/HA (A,B) and GEL (C,D) displaying a good colonization of the 3D scaffolds**. At 21 days (insets), spherical structures (arrows) were present in PDPCs cultured on both tested scaffolds. Similar features were evidenced also in PDL-SCs seeded on GEL/HA (inset Figure 1C).

Morphological analyses showed the ability of cells to colonize the porosity of both tested scaffolds (Figure 2). After 21 days (Figure 2 insets) cells covered the entire surface of the scaffolds, and on PDL-SCs features indicative of induction of mineralization were detected.

### TEM

**Figure 3:**
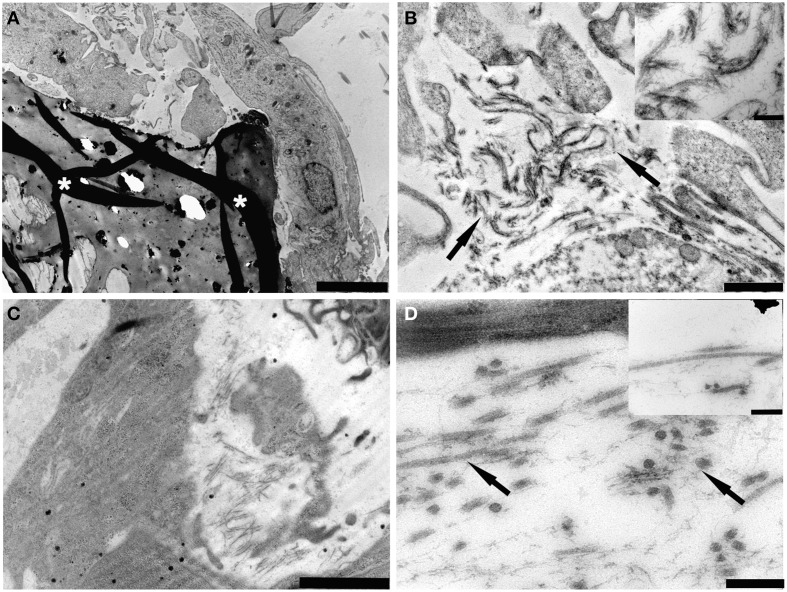
**TEM micrographs of (A) PDL-SCs cultured on gelatin/hydroxyapatite (white asterisks) for 14 days (bar: 5 μm); (B) Fibrils (arrows) resembling the early deposition of extracellular matrix components were detected (bar: 1000 nm)**. The inset shows a detail on collagen type I fibers (bar: 200 nm); **(C)** PDL-MSCs cultured on gelatin/hydroxyapatite for 21 days (bar: 2000 nm); **(D)** A high synthesis of extracellular matrix components (arrows) was observed (bar: 200 nm). The inset shows details on collagen type I fibers detected in the extracellular matrix (bar: 200 nm).

ASCs cultured for 14 days on gelatin scaffold showed a good cell adhesion on the surface of the material (Figure 3A). Nucleus and nucleolus were well-evident (Figure 3A) and a good production of extracellular matrix (ECM) components was observed (Figure 3B).

After 21 days of culture on gelatin scaffolds, cells showed a well-developed rough endoplasmic reticulum (RER) suggesting a high protein synthesis (Figure 3C). ECM matrix was well-noticed (Figure 3D) and fibrillar structures connected to collagen fibers were easily observed (Figure 3D inset).

**Figure 4:**
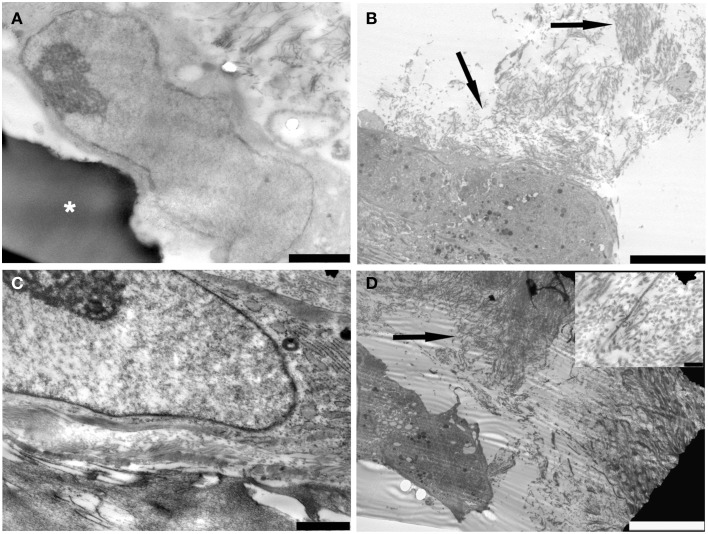
**TEM micrographs of (A) ASCs observed after 14 days of cultured on gelatin scaffold (white asterisks) (bar: 2000 nm); (B) components (arrows) of the extracellular matrix were detected in ASCs cultured for 14 days on scaffold (bar: 10 μm); (C) ASCs cultured on gelatin scaffold for 21 days**. Cells showed a well preserved nucleus and rough endoplasmic reticulum (bar: 1000 nm); **(D)** Several fibrillary structures (arrow) resembling collagen fibers were easily observed in the extracellular matrix (bar: 10 μm). The inset shows a detail on collagen fibers (bar: 200 nm).

TEM images regarding PDL-SCs cultured on gelatin/hydroxyapatite for 14 days demonstrated well-adhered cells on the scaffold surface (Figure 4A). Nucleus and cytoplasmic organelles, such as RER, were well-detected (Figure 4A). Small fibrils resembling the early deposition of collagen type I were observed (Figure 4B). After 21 days of scaffold culture PDL-SCs showed a high synthesis of ECM components with ultrastructurally identified collagen type I fibrils (Figures 4C,D) inset.

### qRT-PCR

**Figure 5:**
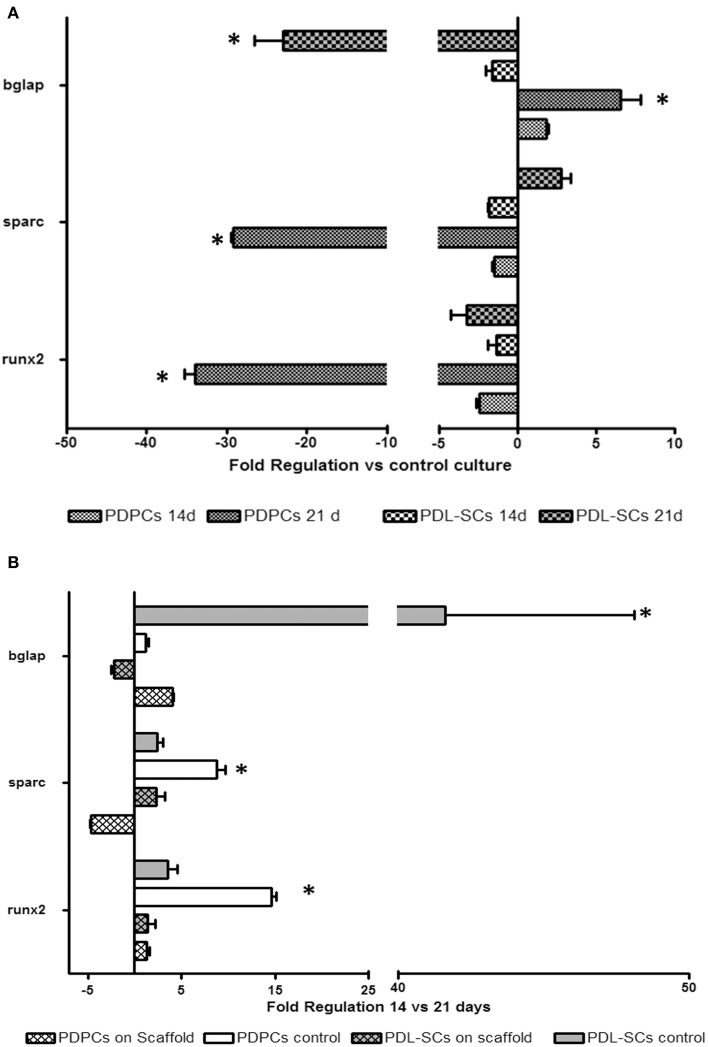
**Histograms depict changes between PDPCs and PDL-SCs mRNA expression of runx2, osteonectin (sparc) and osteocalcin (bglap) observed after culturing cells for 14 and 21 days on GEL/HA with osteogenic differentiating medium. (A)** Fold-changes of PDPCs and PDL-SCs seeded on scaffold with respect to PDPCs and PDL-SCs control cultures (i.e., PDPCs and PDL-SCs in tissue culture plates with osteogenic differentiating medium); **(B)** Fold changes of PDPCs and PDL-SCs seeded on GEL/HA (scaffold) or in tissue control plates (control) at 14 vs. 21 days. Data are expressed as fold-regulation which represents fold-change results in a biologically expressive manner (see Materials and Method section). Statistical differences with relative controls are denoted with an asterisk (^*^p < 0.05).

Comparison of gene expression results in cells seeded onto GEL/HA with control culture in plastic are shown in Figure 5A. Both PDPCs and PDL-SCs showed a reduction in the expression of runx2 after 14 days of culture on GEL/HA, that was detected also after 21 days of culture, being significantly marked in PDPCs. The same trend was observed in PDPCs for osteonectin (sparc) mRNA expression. In PDL-SCs we observed a reduction of mRNA for osteonectin (Fold regulation = −1.5±0.1) after 14 days of culture and its increase (Fold regulation = 2.8 ± 0.6) after 21 days. As far as osteocalcin (bglap) mRNA expression is concerned, PDPCs showed its moderate up regulation after 14 days of culture, that became significantly marked after 21 days (Fold regulation = 6.6 ± 1.2). On the contrary, in PDL-SCs bglap mRNAexpression was down regulated at both time point analyzed with a significant decrease after 21 days of culture.

The assessment of changes in gene expression between 21 and 14 days in cells seeded onto GEL/HA scaffolds compared to those cultured in tissue control plates suggested a different “commitment” of the diverse MSCs populations studied (Figure 5B). In PDPCs we observed a significant up-regulation of bglap on cells seeded on the scaffolds respect to controls. This up-regulation was concomitant with the down regulation of sparc mRNA expression and with a reduced activation of runx2. On the contrary in PDL-SCs seeded on the GEL/HA scaffolds there were slight changes in runx2 and sparc mRNA expression in comparison with controls, whilst we observed a reduction in bglap mRNA expression.

Overall, these results suggested that PDPCs are more committed toward an osteoblastic phenotype compared to PDL-SCs and concomitantly we observed that the scaffold architecture/composition affect osteoblastic differentiation.

**Figure 6:**
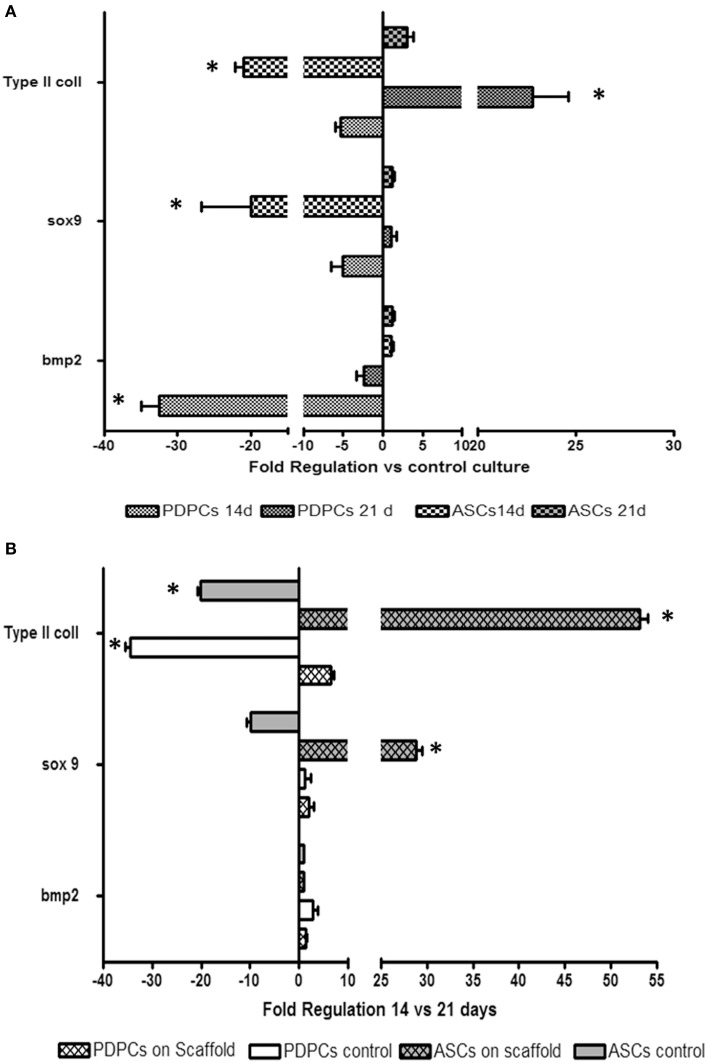
**Histograms depict changes between PDPCs and ASCs mRNA expression of bmp2, Sox 9 and Collagen Type II observed after culturing cells for 14 and 21 days on GEL with chondrogenic differentiating medium. (A)** Fold-changes of PDPCs and ASCs seeded on scaffolds with respect to control cultures (i.e., PDPCs and ASCs in tissue culture plates with chondrogenic differentiating medium); **(B)** Fold changes of PDPCs and ASCs seeded on GEL (scaffold) or in tissue control plates (control) at 14 vs. 21 days. Data are expressed as fold-regulation which represents fold-change results in a biologically expressive manner (see Materials and Method section). Statistical differences with relative controls are denoted with an asterisk (^*^p < 0.05).

As far as cells seeded on GEL scaffold and induced toward a chondrogenic differentiation Bmp2 resulted significantly down regulated in PDPCs, while it remained unchanged in ASCs. Sox9 expression was down regulated in both cell cytotypes at 14 days of culture, whilst it appeared unmodified at 21 days of culture. Results of the comparison of Type II collagen mRNA expression in cells seeded onto the scaffolds with control culture in plastic showed a down regulation of this gene at 14 days that was more marked in ASCs. On the contrary, after 21 days the production of Type II collagen increased for both tested cytotypes (Figure 6A).

The role of a 3D structure in the production of Sox9 and Type II collagen (i.e., chondrogenic commitment) was confirmed by changes in gene expression between 21 and 14 days in cells seeded onto GEL (Figure 6B), in which the increase of mRNA for both genes was observed only in cells seeded on scaffolds.

## Discussion

To restore extensive or complex fracture and/or maxillofacial defects, autograft has been widely used and it is still considered as a gold standard (Dimitriou et al., 2011). Autogenous tissue is endowed of all the basic elements essential for an effective tissue regeneration: it provides cells, extracellular matrix and cytokines (Khan et al., 2005; Pape et al., 2010). Nevertheless, the use of autograft possesses drawbacks in terms of costs, procedure time, patient discomfort and possible complications. Moreover, given the limited availability of autogenous tissues, harvested volume could be insufficient to fill or cover a defect (Zouhary, 2010). To overcome these limitations, a variety of exogenous substitutes has been introduced in clinical practice over the last decades (De Long et al., 2007). Indeed, the presence of cells, which orchestrate the release of growth factors and the maintenance of a stable scaffold is key factor for a positive tissue regeneration as cells play a pivotal role in the healing process (Taba et al., 2005). Both somatic and stem cells can be used: the former can be harvested, cultured and implanted to engineer new tissues. Restrictions in the application of somatic cells are related to the lack of self-renewal capability and limited potency, which are exclusive characteristics of stem cells (Garcia-Godoy and Murray, 2006). Among the latter, MSCs has held a great promise. After their initial detection in bone marrow, several other sources of MSCs were identified, including embryonic tissues (umbilical cord, amnion or placenta), as well as different adult tissues (skin, dental pulp, periosteum and adipose tissue, among others) (Salvolini et al., 2010; Ferretti and Mattioli-Belmonte, 2014a; Lazzarini et al., 2014). Indeed, MSCs of different origin may vary in their ability to proliferate and/or respond to external influences (i.e., microenvironment), this behavior could entail different *in vivo* results after transplantation. In this respect, it is crucial to select the most appropriate MSC type for the healing of different anatomical district injured skeletal tissues. Moreover, to improve bone healing, researchers must develop and/or select a scaffold able to maintain, induce and restore biological functions. Therefore, scaffolds must be evaluated not only for their capability to preserve MSC survival, but also to promote their proliferation and differentiation.

In the present study we compared the functional behavior of MSCs of different origin on two kind of 3D porous scaffolds intended for bone (GEL/HA) or cartilage (GEL) regeneration. In order to mimic microenvironment cell cultures were performed in osteogenic or chondrogenic differentiating medium. MSC harvesting site (periosteum, periodontal ligament or adipose tissue) was selected on the basis of experimental evidences of their possible use in clinical practice.

Periosteum has an inner cambium layer with skeletal progenitor cells that constantly give rise to osteoblasts for appositional bone growth and for cortical bone modeling and remodeling in concert with osteoclasts. The outstanding periosteum property has produced widespread research on the use of periosteum-derived cells (PDPCs) for regenerative approaches. Preclinical studies showing the potential of PDPCs in the treatment of non-healing bone fractures and large bone defects are currently available (Ferretti and Mattioli-Belmonte, 2014a). Upon bone injury, PDPCs tend to initiate endochondral bone formation. This feature seems to be a unique periosteal characteristic, as endosteal or bone marrow lesions heal by intramembranous ossification (Colnot, 2009). This dissimilarity in preferred bone formation is maintained even when cells have been expanded *ex vivo* (van Gastel et al., 2014). Indeed, during post-natal bone repair periosteum is the tissue mainly involved in the generation of tissue-forming progenitors. However, multiple adult stem cell populations can be induced into the osteogenic and/or chondrogenic lineages *in vitro*, and as a consequence used for skeletal regeneration.

Periodontal ligament (PDL) contains cell populations that can differentiate into either cementum-forming cells (cementoblasts) or bone-forming cells (osteoblasts) (Huang et al., 2009). This suggests that PDL contains progenitor cells that maintain periodontal tissue homeostasis and regeneration (Huang et al., 2009). Indeed, PDL-SCs are able to form both soft and hard periodontal tissues *in vivo* and are able to stimulate alveolar bone formation (Seo et al., 2004).

At last, ASCs are easily achievable by lipoaspirates from human adipose tissue (Kim and Heo, 2014). In current literature, confident results of tissue engineering strategies for the reconstruction of large osseous defects in orthopedic and craniofacial surgery are available (Griffin et al., 2014; Marmotti et al., 2014). At present, significant efforts have been made for their application in cartilage regeneration (Griffin et al., 2014; Marmotti et al., 2014).

For these reasons we decided to test PDPCs on both type of scaffolds (with or without HA), using them to check PDL-SCs osteogenic differentiation and ASCs chondrogenic differentiation capability, respectively.

Our results evidenced that PDL-SCs are less osteoblastic committed in comparison with PDPCs and the latter seems to be affected by scaffold structure/composition that accelerates cell differentiation toward osteoblasts. This finding is in agreement with our previous results in which we demonstrated the importance of mechanical properties in the expression of PDPC osteogenic genes and of HA in fastening this event (Mattei et al., 2015). Studies comparing the osteogenic capacity of PDL-SCs with other MSCs sources report conflicting data and this contradiction may be at least in part explained by technical differences between these researches, including cell passage number and osteogenic conditions used. Therefore, as reported by Liu et al. (2008) in an experimental animal model PDL-SCs could be an ideal cellular source for periodontal ligament regeneration and this PDL-SC mediated treatment could in turn recover the heights of alveolar bone. As far as chondrogenic differentiation is concerned, scaffold geometry seemed essential to favor cells chondroblast differentiation, confirming recent observation of other researchers (Chen et al., 2015; Dinescu et al., 2015; Roberts et al., 2015).

In conclusion, the proposed 3D porous scaffolds differing in chemical composition are confirmed as promising candidates for osteochondral tissue regeneration applications. However, in order to achieve a successful cell-based skeletal therapy of different anatomical regions a correct stem cell source selection is mandatory.

## Author contributions

Each author substantially contributed to experimental procedure. In particular MMB planned the whole research and performed SEM analysis; GT performed TEM observation; GO and MF supplied tissue samples for the different cytotypes, VS, SF, and MD executed cell cultures and qRT-PCR, MO was responsible for qRT-PCR analysis. MFa and RD oversaw the whole research. All authors equally and competently contributed to the draft.

## Ethical statement

All patients provided their informed consent to participate in the study in accordance with the Declaration of Helsinki. Since the study did not expose the subjects to any risk and in agreement with the Regione Marche Ethical Committee, instead of a written agreement form, a verbal consent was obtained from all the recruited patients. It was highlighted to all subjects that the tissue used for the study represents the usual surgical discard and that the nature of their participation in the study was entirely voluntary (freedom from coercion or undue influence, real or imagined). Patients had sufficient opportunity to ask questions and consider their choice.

## Funding

This work was supported by FIRB (RBAP10MLK7) and PRIN (2010J8RYS7) grants.

### Conflict of interest statement

The authors declare that the research was conducted in the absence of any commercial or financial relationships that could be construed as a potential conflict of interest.
